# Reactivation of codogenic endogenous retroviral (ERV) envelope genes in human endometrial carcinoma and prestages: Emergence of new molecular targets

**DOI:** 10.18632/oncotarget.679

**Published:** 2012-10-13

**Authors:** Pamela L. Strissel, Matthias Ruebner, Falk Thiel, David Wachter, Arif B. Ekici, Friedericke Wolf, Franziska Thieme, Klemens Ruprecht, Matthias W. Beckmann, Reiner Strick

**Affiliations:** ^1^ University-Clinic Erlangen, Department of Gynecology and Obstetrics, Laboratory for Molecular Medicine, Erlangen, Germany; ^2^ Institute of Pathology, Erlangen, Germany; ^3^ Institute of Human Genetics, Erlangen, Germany; ^4^ Department of Neurology, Charité-University Medicine, Berlin, Germany

**Keywords:** Endometrial carcinoma, hyperplasia, polyp, ERV, Syncytin

## Abstract

Endometrial carcinoma (EnCa) is the most common invasive gynaecologic carcinoma. Over 85% of EnCa are classified as endometrioid, expressing steroid hormone receptors and mostly involving pathological prestages. Human endogenous retroviruses (ERV) are chromosomally integrated genes, account for about 8% of the human genome and are implicated in the etiology of carcinomas. The majority of ERV envelope (env) coding genes are either not present or not consistently represented between common gene expression microarrays. The aim of this study was to analyse the absolute gene expression of all known 21 ERV env genes including 19 codogenic and two env genes with premature stop codons in EnCa, endometrium as well as in hyperplasia and polyps. For EnCa seven env genes had high expression with >200 mol/ng cDNA (e.g. envH1-3, Syncytin-1, envT), two middle >50 mol/ng cDNA (envFc2, erv-3) and 12 low <50 mol/ng cDNA (e.g. Syncytin-2, envV2). Regarding tumor parameters, Syncytin-1 and Syncytin-2 were significantly over-expressed in advanced stage pT2 compared to pT1b. In less differentiated EnCa Syncytin-1, erv-3, envT and envFc2 were significantly over-expressed. Syncytin-1, Syncytin-2 and erv-3 were specific to glandular epithelial cells of polyps, hyperplasia and EnCa using immunohistochemistry. An analysis of 10 patient-matched EnCa with endometrium revealed that the ERV-W 5' long terminal repeat regulating Syncytin-1 was hypomethylated, including the ERE and CRE overlapping MeCP2 sites. Functional analyses showed that 10 env genes were regulated by methylation in EnCa using the RL95-2 cell line. In conclusion, over-expressed env genes could serve as indicators for pathological pre-stages and EnCa.

## INTRODUCTION

Endometrial carcinoma (EnCa) primarily occurs in postmenopausal women and represents the 7^th^ most common malignant disorder. Regarding gynaecologic cancers EnCa is the most common invasive carcinoma. Worldwide 287,100 new carcinoma cases of the corpus uteri were diagnosed in 2008 [1]. For 2012, the National Cancer Institute estimated 47,130 new EnCa cases in the United States of America and 8,010 deaths (http://www.cancer.gov). For Germany, the standardized incidence of EnCa for 2009 was 17.8 per 100,000 women and the mortality 2.9 per 100,000 (www.gekid.de).

The etiology of EnCa entails histological and hormonal differences as well as prior pathological stages. Over 85% of all EnCa cases are histologically classified as endometrioid (type I), mainly expressing steroid hormone receptors. The rare, but more aggressive non-endometrioid EnCa (type II) often lacks steroid hormone receptor expression, and may develop directly from transformed endometrial surface epithelium [2]. The prestage endometrial hyperplasia represents an overgrowth of the endometrium involving both glandular and stromal elements. Estrogens are considered to act as typical tumor promoters in the development from simple to atypical endometrial hyperplasia and then progressing to endometrioid EnCa. Seventy per cent of women with abnormal uterine bleeding have benign causes, whereas 15% have hyperplasias and 15% have a carcinoma [3]. According to the classification of endometrial hyperplasias in simple, complex, with and without atypia, 50% of women with atypical hyperplasia have a concurrent carcinoma and the risk to proceed into a carcinoma is ~30% [3]. Interestingly, in a retrospective study of 538 patients with endometrial hyperplasia, treatment with Progestins led to a decrease of both hyperplasia and progression to a carcinoma [4]. Though yet unclear in the staging of EnCa, endometrial polyps can progress in hyperplasia and carcinoma. For example, endometrial hyperplasia (11.4%) or endometrial carcinoma were found in endometrial polyps (3%) [5] and in another retrospective report 1.8% of women with endometrial polyps had endometrial hyperplasia and 1.3% EnCa [6].

Studies have demonstrated different DNA alterations occurring in EnCa. For example, commonly found changes were increased microsatellite instability due to defects in mismatch repair genes, gene mutations in *PTEN* and *p53* and DNA aneuploidy [7]. Results of our recent publications demonstrated that in disease progression the estrogen receptor (ER) was significantly differentially over expressed in hyperplasia, polyps and in EnCa compared to control endometrium [8, 9]. Several overall gene expression analyses by chip technology showed numerous genes up- or down-regulated in EnCa or between type I and type II EnCa, e.g. over 1,000 genes were found altered between endometrioid, non-endometrioid EnCa and mixed Mullerian tumors [10, 11].

Human Endogenous Retroviruses (ERV) become inherited as a Mendelian gene following retroviral infection and DNA integration of germ line cells. Different ERV gene families constitute about 8% of the human genome and are considered as long terminal repeat (LTR) retrotransposons, in contrast to non-LTR retrotransposons, like LINE and SINE [12, 13]. A ERV provirus consists of the typical retroviral coding regions: gag-pro-pol-env, flanked by 5' and 3' LTR. To date 31 distinct groups and over 100 different ERV families have been found integrated throughout the human chromosomes and represent different copy numbers [13, 14]. For example, some ERV families have high copy numbers, like ERV-H (660 copies), whereas some exhibit low copy numbers, like ERV-FRD and ERV–R (each 15 copies) and ERV-Fc (6 copies) [14, 15]. Fifty copies of the ERV-E family member 4-1 were found integrated at 30 chromosomal sites [16]. The ERV-W family represent a total of 140 provirus and retrosequences throughout the genome [17].

ERV genes can promote homologous and non-homologous recombination and are initiators of new mutations [18, 19]. Therefore, ERVs contribute to genome wide instability, most likely contributing in tumor initiation and progression [13]. Regardless of millions of years since integration into the genome, some ERV genes still have an open reading frame (ORF) and protein expression. Table 1 shows an up to date summary of 19 different fully coding ERV env genes and two ERV env genes with stop codons from 11 different ERV families [20] (Table 1). Although envE of ERV-E4-1 is not a full length env, due to a stop codon after 428 amino acids [21], antibodies detected an envE protein in control and tumor tissues [22]. Furthermore, envW2 on chromosome Xq22 was demonstrated as transcribed, but harboured an N-terminal stop-codon after 117 bp [23]. EnvW2 has a DNA similarity of 93.5% to the ERV-W env gene on chromosome 7q21.2, called Syncytin-1. In addition, erv-3 (envR), a single nucleotide polymorphism (SNP) has been predicted to have an incidence of 1% homozygosity in the caucasian population, translating into a physiological stop-codon after 182 amino acids [24].

**Table 1:** Chromosomal localization and amino acid length of all analyzed 21 env genes HERV-K1-6 and HERV-H1-3 numbering is according to de Parseval et al. [20]; the three envH1-3 and the six envK1-6 were each analysed with one primer pair. EnvE has a stop codon after 428 amino acids (aa) and envW2 has a stop codon after 38 amino acids or 117 bp. {ERV}= according to nomenclature of Mayer et al. [69]

	HERV {ERV}*	env	chromosome	Acc.#	coding {aa}
1	F(c)2 {ERVFC1-1}	envFc2	7q36.2	AC016222	528
2	F(c)1	envFc1	Xq21.33	AL354685	584
3	FRD {ERVFRD-1}	Syncytin-2	6p24.1	AL136139	538
4	H1	envH1 (p62)	2q24.3	AJ289709	585
5	H2	envH2 (p60)	3q26	AJ289710	563
6	H3	envH3 (p59)	2q24.1	AJ289711	555
7	K1 HERV-K74261 {ERVK-21}	envK1	12q14.1	AC074261.3	698
8	K2 HML-2.HOM / K108 {ERVK-6}	envK2	7p22.1	AC072054	699
9	K3 HERV-K17833 C19 {ERVK-19}	envK3	19q12	Y17833	694
10	K4 HML-2 (K109) {ERVK-9}	envK4	6q14.1	AF164615	698
11	K5 HERV-K113 {ERVK-22}	envK5	19p13.11	AY037928.1	699
12	K6 HERV-K115 {ERVK-8}	envK6	8p23.1	AY037929.1	699
13	P(b)	Syncytin-3	14q32.12	ABB52637.1	665
14	R {ERV3-1}	erv-3	7q11.21	AC073210	604
15	R(b) {ERVPABLB-1}	envRb	3p24.3	AC093488	514
16	T {ERVS71-1}	envT	19p13.11	AC078899	626
17	V1 {ERVV-1}	envV1	19q13.41	NM_152473	477
18	V2 {ERVV-2}	envV2	19q13.41	NM_001191055	535
19	W {ERVW-1}	Syncytin-1	7q21.2	AC000064	538
20	E4-1	envE	19q12	AB062274.1	428
21	W2 {ERVW-2}	envW2	Xq22.3	FN689795	(38)

Interestingly, some env genes are expressed in normal tissues and associated with positive and beneficial physiological functions, like Syncytin-1, which is essential for placentogenesis [20, 25-27]. After binding to cellular receptors some retroviral env genes are responsible for cell-cell fusions, like fusions of human placental villous trophoblasts into a multinucleated syncytiotrophoblast responsible for gas and nutrient exchange [25, 27]. To date, three ERV env genes, Syncytin-1, env-FRD (Syncytin-2) and env-Pb (or Syncytin-3) have been demonstrated as promoting cell-cell fusions *in vitro* [25, 26, 28-30]. Interestingly, env genes were also found significantly over-expressed in primary tumors or cancer cell lines including envK (HML2) in germ cell tumor lines [31], melanoma [32], breast cancer [33] and ovarian cancer tissue [34]; Syncytin-1 over expression was found in breast cancer tissue [35], EnCa [8, 9], colon carcinoma [36], and leukemia and lymphoma [37]. An involvement of Syncytin-1 in cancer cell-cell fusions was demonstrated *in vitro* for human rhabdomyosarcoma cell line [25], choriocarcinoma cell line [26], breast cancer [35] and EnCa [8]. In general, transposable elements including ERV genes are silenced preferentially by methylation. Evidences for variable methylation of the LTR were found for ERV-K [38], ERV-E [39], ERV-W and ERV-FRD [40, 41].

The majority of ERV env coding genes are either not present or not consistently represented between common gene expression microarrays. ERV env genes or flanking non-coding regions represented on gene expression microarrays from Affymetrix or Agilent by gene and sequence search to date were Syncytin-1 (ervwe1), Syncytin-2 (ERV-FRD), envK2 (ERV-K6), erv-3 (erv3-1), envV1 (ERVV-1) and envFc2 (ERVFC1-1). However, most primers of these env genes for microarrays were designed outside the codogenic regions. Thus, previous gene microarray studies did not have a complete and clear representation of all codogenic ERV env genes. In order to test, if ERV env genes play a significant role in EnCa, endometrial polyps and hyperplasia, we cloned all known 21 codogenic ERV envelope (env) genes including two env genes with premature stop codons to quantify their expression levels. In addition, we immunolocalized Syncytin-1, Syncytin-2 and erv-3 expression in EnCa and prestages as well as analyzed the entire ERV-W 5' LTR for methylation in patient matched tumor and control tissues.

## RESULTS

### ERV env genes are over-expressed in EnCa, hyperplasia and polyps and significantly correlate with tumor staging

**Figure 1:**
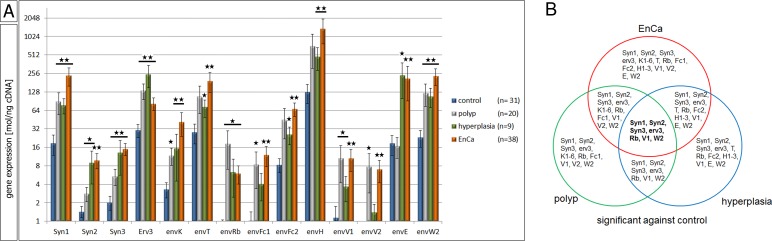
QPCR of 21 ERV env genes in normal and pathological endometrial tissues A: Gene expression analysis by qPCR of control endometrium (n=31), endometrial polyp (n=20), endometrial hyperplasia (n=9) and EnCa (n=38) for 21 ERV env genes. *= P:<0.05 and **= P:<0.005. B: Venn blot of all significant env genes is shown from a comparison of all tissues compared to control endometrium. The intersection represents 7 env genes significantly increased in expression in EnCa and prestages (in bold). Syn= Syncytin.

To compare the expression of ERV env genes in EnCa, pathological prestages and control endometrium we performed full quantitative gene analyses of 21 different codogenic ERV env genes including two env genes with premature stop codons. Strikingly, all 21 ERV env genes were statistically significantly higher expressed in EnCa (a total of 2,511.2 mol/ng cDNA without s.e.m.) compared to control endometrium (total of 263.3 mol/ng cDNA without s.e.m.) (Fig 1A, suppl. Table 2). Most of the ERV env genes were also significantly higher expressed in EnCa compared to polyps and / or hyperplasia, except for erv-3, envRb, envV2 and envE. Comparing prestages to control endometrium, apart from a few non-significantly expressed env genes in polyps (envT, envFc2, envH, envE) and hyperplasia (envK, envFc1, envV2) all other ERV env genes were statistically significantly higher (Fig. 1A, suppl. Table 2). Interestingly, we also observed three different levels of ERV env gene expression among the pathological tissue cohorts. For example in EnCa, env genes with a high expression of >200 mol/ng cDNA were Syncytin-1, envT, envH, envE and envW2 (supple. Table 2). Particularly, Syncytin-1 (236.76 mol/ng cDNA, P=6.72 E-8) and envW2 (234.8 mol/ng cDNA, P=8.03E-7) showed similar levels of expression. ERV-H1-3 env genes, which included three different codogenic envH genes (p62, p60 and p59), were the highest expressed among all tissue cohorts (1,378 mol/ng cDNA for EnCa, P=3.99 E-5) (Table 1, supple. Table 2). On the other hand erv-3 (82.95 mol/ng cDNA, P=4.10 E-5) and envFc2 (67.80 mol/ng cDNA, P=8.06 E-6) were moderately expressed in EnCa. Lower expressed env genes of 50 mol/ng cDNA or less, which were statistically significantly higher in expression for EnCa and partly for hyperplasia and polyps when compared to normal endometrium were Syncytin-2, Syncytin-3, envK1-6, envRb, envFc1, envV1 and envV2 (Fig. 1A, suppl. Table 2).

Regarding erv-3, which showed significantly higher expression in polyps, hyperplasia and EnCa, we also asked the question, if the expression of erv-3 in EnCa was significantly linked with a previously rare published nonsense mutation. Using our designed SNP-assay, results showed no significant differences between EnCa and endometrium controls, where 85.4 % of tumors were homozygous wild-type, 14.6% heterozygous for the nonsense mutation and 0% of tumors were homozygous mutant (data not shown). Thus, all tumors and controls would produce a full-length erv-3 protein.

**Figure 2:**
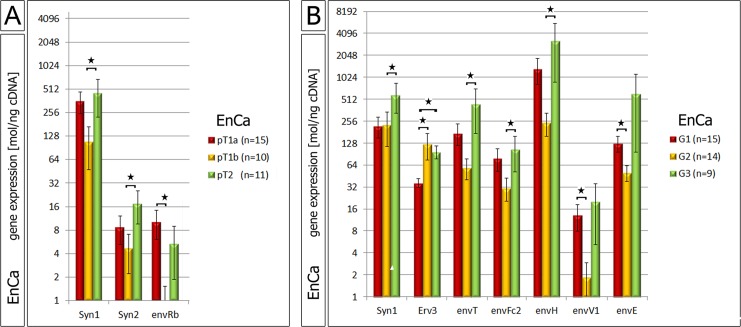
Correlation of ERV env genes with EnCa staging and histology A: Significant gene expression differences of Syncytin-1, Syncytin-2 (Syn) and envRb according to different EnCa staging (pT). B: Significant gene expression differences of env genes according to differentiation of EnCa (G1-G3). Statistically significance values are shown for both pT staging and G1-3 tumor grading as *= P:<0.05.

A statistical three way analyses was performed between all significant env genes (polyp, hyperplasia and EnCa vs. control endometrium). Among all pathological tissue cohorts the six common significant env genes (“intersection”) were Syncytin-1, Syncytin-2, Syncytin-3, erv-3, envRb and envV1 (Venn diagram, Fig. 1B). Furthermore, the gene expression of some of the env genes significantly correlated with EnCa tumor stage (TNM) and histological grading (G1-G3) (Fig. 2, suppl. Table 3). For example, both Syncytin-1 and Syncytin-2 expression significantly correlated with a tumor progression from pT1b to pT2 (Fig. 2A). Analysing histological grading (G) stages with env gene expression showed that Syncytin-1 significantly increased in expression from G2 to the more undifferentiated G3 stage; erv-3 higher expression was also found in G2 and G3 when compared to the differentiated G1 stage; envT, envFc2 and envH showed an increase of expression from G2 to G3, and envV1 and envE decreased in expression from G1 to G2 (Fig. 2B). Among the ERV-env genes only erv-3 was significantly differentially expressed between stage G1 and G3 and only Syncytin-1 significantly correlated with progression of both pT1b to pT2 and G2 to G3 tumor grading.

### Immunolocalization of Syncytin-1, Syncytin-2 and erv-3 in normal endometrium, polyps, hyperplasia and EnCa

**Figure 3:**
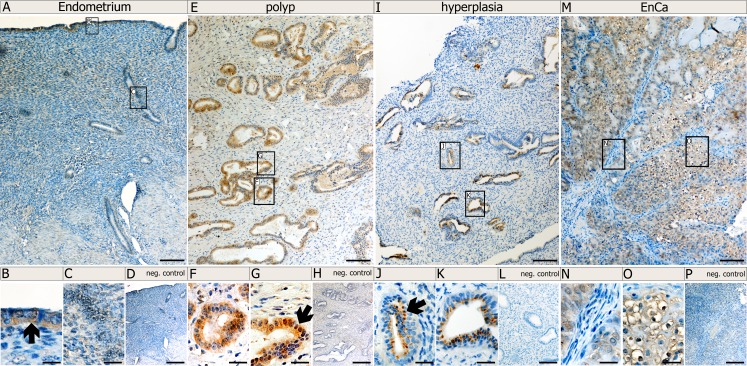
Protein localization of Syncytin-1 in normal and pathological endometrial tissues Immunohistochemistry (IHC) using Syncytin-1 antibody and paraffin embedded tissues from a control endometrium (A-D); a polyp (E-H), and a complex hyperplasia with atypia (I-L) and an EnCa (M-P). Top row of images (A,E, I and M) represent low magnification (bar=100μm). Bottom row of images represent a high magnification (bar=25μm) of the boxed regions from the low magnification representations. Arrows point to specific morphological structures positive for Syncytin-1 protein expression (brown staining): B: surface epithelium; F, G: epithelial gland cells from a polyp, J, K: epithelial glandular cells from a complex hyperplasia tissue with atypia and showing Syncytin-1 cytosolic protein expression as “punctate” foci (“Golgi-like”) localizing para-nuclear or apically at the membrane; N: EnCa stroma (blue) negative for Syncytin-1 expression and O: Syncytin-1 positive expression in tumor epithelial cells (note positive membrane staining). Negative (neg.) controls (D, H, L, P) represent IHC without Syncytin-1 antibody, but with the secondary antibody.

**Figure 4:**
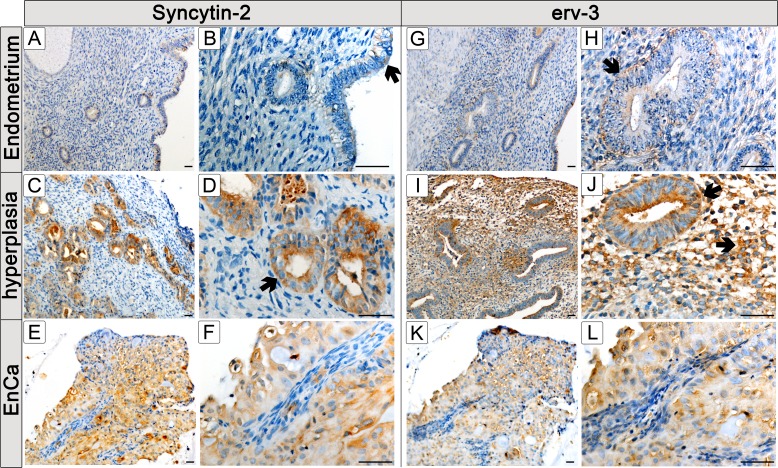
Protein localization of Syncytin-2 and erv-3 in normal and pathological endometrial tissues Immunohistochemistry (IHC) using Syncytin-2 and erv-3 antibodies and paraffin embedded tissues from a control endometrium (A,B,G,H); a complex hyperplasia with atypia (C,D,I,J) and EnCa (E,F,K,L). Syncytin-2 and erv-3 left column (low magnification) and right column (high magnification) of microscope pictures are shown (bar=50μm). Arrows point to specific morphological structures positive for Syncytin-2 and erv-3 protein expression (brown staining): B: surface epithelium showing a weak focal Syncytin-2 staining of a ciliated tubal epithelial (mucous producing cell); D: Syncytin-2 positive expression in epithelial gland from a complex hyperplasia tissue with atypia; F: Syncytin-2 expression is negative in EnCa stroma (blue) but positive in epithelial glandular tumor cells (note both cytolosic and membrane staining). For erv-3 protein expression arrows show: H: positive expression in epithelial glandular cells of normal epithelium, J: erv-3 positive expression in epithelial gland and stroma cells from a complex hyperplasia tissue with atypia; L: erv-3 expression is negative in EnCa stroma (blue) but positive in epithelial glandular tumor cells.

Since expression of Syncytin-1, Syncytin-2 and erv-3 significantly associated with EnCa tumor stage (TNM) and histological grading (G1-G3), we performed IHC to co-localize these env proteins in tissues. Control endometrium (n=2) showed a weak cytosolic staining of surface and glandular epithelial cells for Syncytin-1 and erv-3 (Fig. 3,4). Syncytin-2 demonstrated a very weak focal protein expression in surface ciliated tubal type epithelial (mucous producing) cells and in some glandular epithelial cells (Fig. 4) All three ERV-env genes demonstrated a moderate to strong protein expression in the cytosolic (Syncytin-1, Syncytin-2 and erv-3) and membrane regions (Syncytin-1, Syncytin-2) of EnCa epithelial glandular cells with no expression in the tumor stroma (n=2) (Fig. 3,4). Additionally, Syncytin-2 protein expression localized to endothelial cells surrounding blood vessels in tumor tissues (data not shown). In simple hyperplasia tissues without atypia, a weak to moderate protein expression of Syncytin-1 and Syncytin-2, respectively, was noted focally in both surface epithelial ciliated cells and in epithelial glandular cells, but was negative in the stroma (n=2) (data not shown). In complex hyperplasia with atypia (n=2), Syncytin-1 and Syncytin-2 expression was moderate to strong in epithelial cells. Additionally, only Syncytin-1 showed strong “punctate” foci in the cytosol at the membrane lining of epithelial glandular cells, which localized para-nuclear or apically (“Golgi-like”) (Fig. 3I-K). Interestingly, erv-3 protein expression was moderate to strong and localized in the cytosol, but also showed a very prominent apical glandular staining for both simple (n=2) and complex hyperplasia with atypia (n=2) (Fig. 4 and data not shown). In contrast to both Syncytin-1 and Syncytin-2, erv-3 additionally demonstrated a moderate to strong protein expression throughout the stroma of all hyperplasia tissues. Regarding polyps (n=2) erv-3 protein expression was moderate in both stroma and glandular epithelial cells, whereas Syncytin-1 and Syncytin-2 were weak to moderately expressed in the cytosol of glandular epithelial cells (Fig. 3 and data not shown).

### ERV-W 5' LTR promoter region in EnCa tissues was regulated by hypomethylation

**Figure 5:**
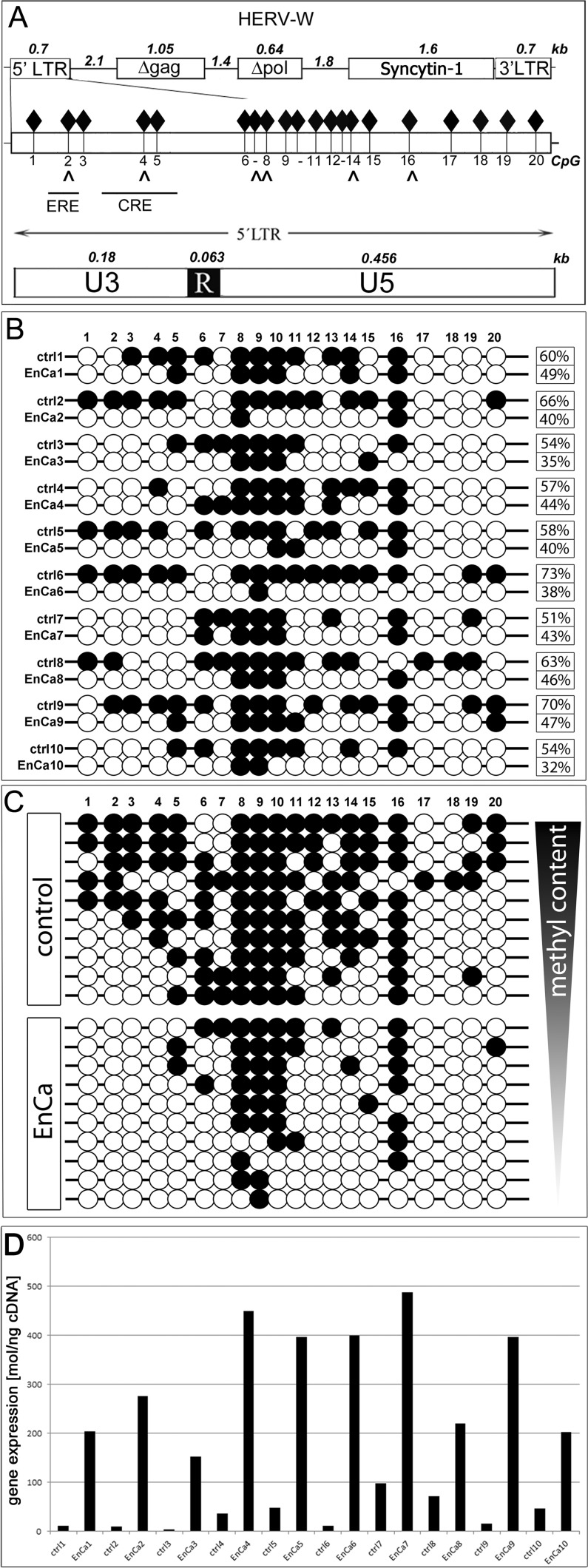
DNA methylation analysis of ERV-W 5'LTR A: Top. Schematic ERV-W provirus with 5'LTR-gag-pol-env (=Syncytin-1) -3'LTR. Numbers represent distances in kb. D= non-codogenic genes gag and pol. Middle graph shows the entire 5'LTR with all 20 CpG (black diamonds) and directly below are arrows which correlate with specific CpG within MeCP2 sites along with the overlapping ERE and CRE DNA elements. Also represented at the bottom graph are the components of the 5'LTR (U3-R-U5). Note that the U3 represents the promoter region containing CpG1-5. B: Analysis of each of the 20 CpG for EnCa and patient matched control endometrium (1-10). Numbers at the right represent the overall methylation level in per cent. Open circle = CpG less than 60% methylated, closed circle = >60% methylation. C: Schematic of control and EnCa organized according to decreasing methylation levels of patient tissues 1-10. D. Graph represents qPCR gene expression values in mol/ng cDNA of Syncytin-1 for each patient modified control endometrium and EnCa patient tissues from B & C. Fold induction of Syncytin-1 were for patient #1: 17-fold, #2: 27.6-fold, #3: 38-fold, #4: 12.2-fold. #5: 8.2-fold, #6: 37-fold, #7: 5-fold, #8: 3.1-fold, #9: 25.5-fold, #10: 4.4-fold.

In order to identify a molecular mechanism to explain ERV env gene induction in tumors, specifically Syncytin-1, we compared the methylation pattern of the ERV-W 5' LTR promoter region of 10 EnCa with 10 patient matched endometrium (Fig. 5). The 5'LTR promoter region of ERV-W contains 20 CpGs and a variety of regulatory elements important for placental development especially the cAMP response element (CRE) (Fig. 5A). In addition, we previously identified a functional estrogen receptor element (ERE) binding site in the ERV-W 5'LTR [8]. Taken together, we reasoned that if de-methylation occurred during tumorigenesis this could lead to chromatin opening and availability of transcription factor binding sites, thus inducing Syncytin-1 expression via the CRE and the ERE.

Following bisulfite-treatment of genomic DNA from EnCa (n=10) and corresponding patient matched tumor-free endometrium tissues (n=10), we analysed all 20 CpGs of the ERV-W 5'LTR by sequencing a total of 120 clones (EnCa) and 103 clones (endometrium). Although each patient tissue pair had a distinct methylation profile, we observed prominent de-methylation at specific CpG-residues, especially at CpG1-5 (within the promoter region of U3), CpG12-15 and CpG17-19 among all 10 EnCa tissues (Fig. 5B). CpG2 harbours the promoter binding sites of both the ERE and the methylation specific binding protein MeCP2; whereas CpG4 overlapped with a MeCP2 binding site and the CRE, GATA-1, GATA-2 transcription factor binding sites. Furthermore CpG8, 9 or 10 was always methylated in the 10 EnCa and CpG16 was found methylated in 7 of 10 EnCa, where putative MeCP2 sites were found (CpG8,16) (Fig.5B). Calculating the total per cent methylation change of CpG1-20 for each patient tissue pair resulted in differences (Fig. 5B). For example, patients #2, #6, #9 and #10 showed a >20% overall change in de-methylation compared to matched endometrium. Correlating qPCR gene expression data for each patient matched normal endometrium and EnCa pair showed an induction of Syncytin-1 in EnCa and associated with a de-methylated U3. The highest Syncytin-1 induction in EnCa had patient #3 and #6 (38- and 37-fold) compared to matched control (Fig. 5D). Furthermore, the combined methylation for all 10 tissue pairs showed an overall change of 16 out of 20 CpGs or a total of 60% methylation in the patient matched endometrium and 41% in the EnCa. Therefore, the overall mean de-methylation change occurring in patient matched endometrium to EnCa was 19%.

**Figure 6:**
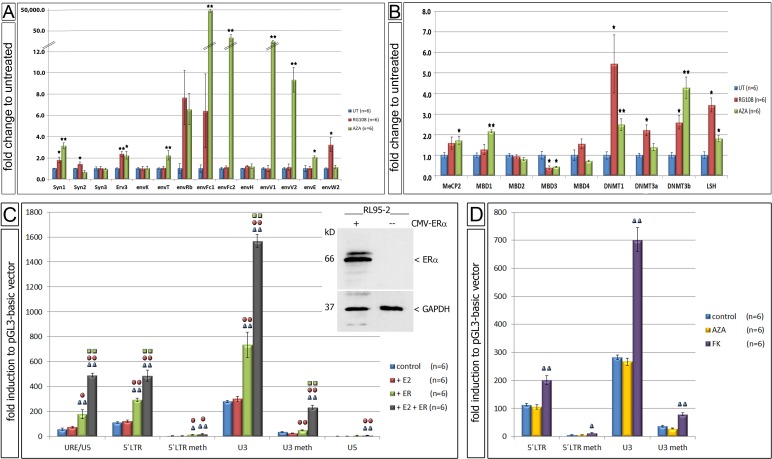
Functional cell culture studies using demethylation agents A. Gene expression differences of env genes in fold change after treatment with AzadC (AZA) (n=6) and RG108 (n=6). B: Gene expression differences of 9 methyl-DNA binding proteins after treatment with AzadC (AZA) (n=6) and RG108 (n=6) (untreated = UT). * P:<0.05 and **= P:<0.005. C: Changes of luciferase activity using transfection of different cloned plasmids of ERV-W 5'LTR and estradiol (E2) and ERα in RL95-2-ERα(-) (n=6). Insert shows immunoblot for ERα and GAPDH of RL95-2-ERα(-) cells with and without transfection of CMV-ERα. D: Changes of luciferase activity of different ERV-W 5'LTR after AzadC (AZA) (n=6) and FK treatment (n=6) of RL95-2-ERα(-). Meth= prior methylated DNA. Symbols above each column represents the statistical significance to each experiment; e.g. blue symbol = significant to control. * P:<0.05 and **= P:<0.005.

Since the 5' LTR of ERV-W showed significant changes in de-methylation we performed functional cell culture studies using methylation inhibitors. Upon treatment of the EnCa cell line RL95-2-ERα(-) with AzadC or RG108, a significant increase of expression of eight and four specific ERV-env genes was detected, respectively (Fig. 6A). Interestingly, both inhibitors showed some similarities and clear differences. AzadC is a cytidine analogue, which first leads to a rapid loss of the natural cytosine base in the DNA and second interferes with DNA methylation activity due to DNMT enzymes becoming irreversibly bound to AzadC residues. RG108 is a specific covalent DNMT1 competitor resulting in enzyme inhibition. Results showed that both AzadC and RG108 significantly induced Syncytin-1 (3.2- and 1.8-fold, respectively) and erv-3 (2.2- and 2.4-fold, respectively). AzadC significantly induced envT (2.2-fold), envFc1 (49,986.8-fold), envFc2 (33.1-fold), envV1 (15.1-fold), envV2 (9.4-fold) and envE (2.1-fold), whereas RG108 significantly induced Syncytin-2 (1.5-fold), envH1-3 (1.2-fold) and envW2 (3.3-fold) (Fig. 6A). For comparison, in a second EnCa cell line, HEC1A-ERα(-) AzadC also induced expression of the following ERV-env genes: Syncytin-1, Syncytin-3, erv-3, envT, envFc1, envFc2, env-H1-3 and envV1 (n=2, data not shown). Furthermore, we tested the same RL95-2 EnCa samples treated with AzadC or RG108 to determine, if nine independent regulatory methylation genes were also altered in expression. Interestingly, both AzadC and RG108 resulted in a significant increase of DNMT1, DNMT3b and LSH gene expression, but in contrast MBD3 expression was significantly reduced. AzadC alone caused a significant increase of MBD1 and MeCP2 expression, whereas DNMT3a was induced with RG108 (Fig. 6B). These results showed that in the presence of de-methylation agents regulatory methylation genes also significantly changed in expression.

### ERV-W LTR regulation of luciferase activity in EnCa cell lines

Following the discovery that de-methylation of the ERV-W 5'LTR was linked with increased Syncytin-1 expression after treatment of EnCa cell lines with AzadC and RG108, we conducted luciferase transfection studies using four different ERV-W-LTR-luciferase constructs. We tested the idea that non-methylated versus *in-vitro* methylated ERV-W-LTR-luciferase constructs would help to prove, if the CRE and the ERE in the U3 region are regulated by methylation in EnCa cells. Furthermore, we reasoned that co-transfection of a CMV-ERα expressing construct into a RL95-2-ERα(-) cell line would help to unravel the estrogen regulation of ERV-W 5'LTR.

Following E2 treatment of CMV-ERα co-transfected RL95-2-ERα(-) cells a significant up-regulation of luciferase activity for all un-methylated luciferase vectors containing the ERV-W-URE/U3/R/U5, -5'LTR (U3/R/U5) and the U3 regions was found (Fig. 6C and insert). The ERV-W-U3 harbouring the ERE containing luciferase vector showed the highest significant induction among all ERV-W-luciferase vectors. Interestingly, co-transfections of both ERV-W-luciferase and the CMV-ERα vector without E2 also showed a significant luciferase induction compared to controls, which we attributed to E2 concentrations in the cell culture medium (FCS). In contrast, CMV-ERα co-transfections with the ERV-W-U5 luciferase vector, which does not contain the ERE, showed a slight, but significant luciferase induction, suggesting the possibility of a diverged ERE element in the ERV-W-U5. On the other hand, RL95-2-ERα(-) cells treated with E2 and co-transfected with *in vitro* methylated ERV-W-5'LTR or ERV-W-U3 luciferase vectors and the CMV-ERα vector resulted in a significant decrease of luciferase activity (Fig. 6D). Taken together, the above results proved that E2 stimulation of RL95-2-ERα(-) cells transfected with ERα led to transcriptional activation most likely due to the ERE in the ERV-W-U3. In addition, our findings support that the ERE of ERV-W-U3 is regulated by methylation.

Following FK treatment of RL95-2-ERα(-) cells transfected with the ERV-W-5'LTR and ERV-W-U3 luciferase vectors demonstrated a significant luciferase induction for both vectors (2.0-fold and 701.8-fold, respectively) (Fig. 6D). Upon transfection of *in vitro* methylated ERV-W-5'LTR and ERV-W-U3 vectors, luciferase induction was significantly decreased, supporting that the CRE is functional in EnCa cells and regulated by methylation. A control experiment was performed treating transfected cells with AzadC and then analyzing for luciferase activity. As expected no differences in luciferase activity was observed compared to control, demonstrating that our *in vitro* methylation was specific since these luciferase vectors did not replicate in cells.

## DISCUSSION

In order to understand the relevance of ERV-env genes in EnCa and prior endometrial pathogenic prestages, we quantified the copy numbers of 21 env genes. Since these env genes are either not represented or not accurately represented on gene expression microarrays our study brings forth new information about the expression of ERV env genes and their hierarchical distribution in EnCa and pathological prestages. In addition, our study highlights the importance of ERV env genes in tumor staging and grading as well as their cellular localization in these tissues and the role of the ERV-W ERE.

Although the ERV-H family is one of the most common ERVs in the human genome, only three (p59, p60, p62) out of 100 env-containing proviruses have a full length ORF transcribed [42-45] (Table 1). In a combined qPCR all three ERV-H env p59, p60 and p62 genes represented the highest transcript levels of all ERV env tested for control endometrium, polyps, hyperplasia and EnCa. ERV-H transcripts have been previously detected in placenta and in a variety of tumor cell lines [46]. An analysis of all three ERV-H env genes in control human tissues showed only for testis relatively low transcript levels [20]. The high ERV-H1-3 env expression noted in our study in EnCa and prestages proposes a functional role in both benign and malignant endometrium. One function could be a role in immunosuppression, which was shown for ERV-H env-p62 in engrafted mice [47].

The env gene of ERV-R (erv-3) has been shown to be expressed in most tissues, like testis, skin, thymus and placenta and in various carcinomas, like glioma, breast, Wilm's tumor [20, 48]. Using our three way statistical analyses, erv-3 was one of the most significant genes identified in the intersection of all three cohorts along with Syncytin-1, Syncytin-2, Syncytin-3, envRb and envV1. Furthermore, our genotyping of an erv-3 stop-codon [24] showed a similar distribution of wild-type (85.4% endometrium and EnCa) and heterozygote (14.6 % endometrium and EnCa). These findings indicate that the over-expressed erv-3 gene in EnCa cells represented a full length protein in the tumor. In contrast to the three known fusogenic ERV-env proteins (Syncytin-1, Syncytin-2 and Syncytin-3), erv-3 is considered a cytoplasmic protein due to the lack of a leader sequence, a membrane spanning domain and a fusion peptide [49]. Interestingly, we observed a mean 3-fold higher gene expression in endometrial hyperplasia compared to EnCa, which was reflected by a moderate to strong cytosolic protein expression localizing to epithelial glandular and stromal cells (Fig. 4). Similar to hyperplasia, endometrial polyps also showed prominent expression of cytosolic epithelial glandular and stroma positive cells. Our data support a linkage of expression between seven ERV env genes (Syncytin-1, erv-3, envT, envFc2, envH, envV1, envE) in tumor progression to a more undifferentiated G3 state. Taken together, the above findings support an early role of erv-3 in endometrial staging and possibly in tumor differentiation. Interestingly, there is some support in the literature of erv-3 being important for differentiation, for example, over-expressing erv-3 vectors controlled hormone production (b-hCG), caused a cell cycle arrest, increased differentiation-related changes and cell fusion in the choriocarcinoma BeWo line [50].

Other significantly over-expressed ERV-env genes in EnCa and endometrial prestages, like envE and envK have also been identified in a variety of tissues and specific carcinomas [20, 22, 33, 34, 51-55]. Strong envK protein expression co-localized to epithelial breast tumor cells and correlated with ductal carcinoma in situ, invasive ductal carcinoma, lymph node metastasis and was further implicated in triggering an antigen immune response [54, 56]. Surprisingly, our simultaneously qPCR of all six envK only resulted in low to medium expression levels less than 50 mol/ng cDNA, which were 30- to 60-fold lower than for the ERV-H1-3 env genes. Although, we observed a stepwise increase in envK1-6 expression from control endometrium to EnCa, we found no statistical association of envK1-6 expression associated with tumor staging and growth patterns.

Where the newly identified envV1 and envV2 genes were specific to placental tissues, Syncytin-3 was found ubiquitously expressed being especially high in bone marrow and testis using qPCR [28]. Although Syncytin-2 and Syncytin-3 like Syncytin-1 were proven to be cell fusogenic using ex-vivo assays, and Syncytin-2 associated with immunosuppressive capabilities, no report to date is known about Syncytin-2 and Syncytin-3 expression in primary carcinomas [28, 57]. While we classified Syncytin-2 and Syncytin-3 as lowly expressed genes (<15 mol/ng cDNA) among our cohorts, we observed a significant step-wise increase from control endometrium to hyperplasia and EnCa. Furthermore, our finding that Syncytin-2 co-localized to epithelial EnCa tumor cells and correlated significantly with pT1b to pT2 tumor staging, supports a possible role of Syncytin-2 in tumor growth. Additionally, the co-localisation of Syncytin-2 around blood vessels in tumor sections may be reminiscent of the placenta syncytiotrophoblast as a maternal / fetal blood barrier and could support tumor immunosuppressive activity [58].

Although there is a wealth of knowledge regarding the molecular process of methylation of gene loci, the mechanism of how DNA de-methylation is carried out during embryonic development and somatic cells is on-going. It is hypothesized that CpG-methylation of promoters is associated with the inability of transcription factor binding leading to a loss of transcriptional activity and converse for CpG-de-methylation [59]. Multiple genes are known to be differentially regulated by methylation in EnCa. Besides an over 50% mutation rate of PTEN in EnCa, PTEN was also methylated in 18% of EnCa [60]. EnCa associated with increased microsatellite instability is most likely due to hyper-methylation of DNA mismatch repair genes [61]. Especially during placentogenesis ERV expression appeared to be tightly regulated by fluctuations of methylation, which could represent a model for the regulation of ERVs in carcinogenesis. For example, methylation analysis of the ERV-W LTR-U3 region demonstrated a hypo-methylated state during the first trimester and a methylated state during the last trimester [40]. In contrast, the ERV-FRD LTR-U3 and ERV-R LTR-U3 regions were hypo- and intermediately methylated, respectively, during all trimesters [40]. Matouskova et al. [41] analysed only 5 of the 20 CpG sites in the 5'LTR of ERV-W and found a more hypo-methylated state in two term placentas, but in contrast a hyper-methylated state in fibroblasts and breast carcinoma. Methylation analysis of the ERV-K (HML-2) 5'LTR-U3 region and the UTR showed that CpG-hypo-methylation was linked with transcriptional activity in melanoma cell lines [62]. However, in contrast some germ cell tumor lines showed that CpG-methylation cannot solely be responsible for transcriptional regulation of ERV-K (HML-2) [38]. In testicular cancer methylation of the 5'-LTR-U3 region of ERV-W, ERV-FRD and ERV-H decreased compared to control tissue, but in contrast the ERV-E loci remained methylated like control tissue [51]. Our analysis of the entire ERV-W 5'LTR (U3/R/U5) showed a significant hypo-methylation of 14 of 20 CpGs in EnCa, reducing the overall ERV-W 5'LTR methylation degree for patient matched endometrium to EnCa by 19%. The decline of ERV-W 5'LTR methylation could be one molecular mechanism responsible for increased expression of Syncytin-1 in EnCa. Additional support for regulation of ERV-W by methylation was observed from our luciferase studies using EnCa cell lines, where a shut-down of luciferase expression occurred upon methylation of ERV-W 5'LTR containing vectors. Furthermore, AzadC or RG108 treated EnCa cell lines helped to identify other ERV-env genes regulated by methylation. For example, these de-methylation agents significantly increased expression of ten other ERV-env genes as well as seven genes involved in regulation of methylation in EnCa cells (Fig. 6A,B). Therefore, we hypothesize that aberrant hypo-methylation of Syncytin-1 and other env genes leads to reactivation of expression where these env genes could possibly function together in early endometrial prestages and EnCa.

We propose that Syncytin-1 plays a leading role in progression to EnCa and has clinical and pathological implications. In concert with other ERV-env genes Syncytin-1 represented: 1) the single highest expressed gene in EnCa; 2) was among the most significant six env genes in a comparison of all three cohorts; 3) along with Syncytin-2 and erv-3 co-localized to endometrial polyp, hyperplastic and tumor epithelial cells pointing to a functional role in this cell type; 4) was significantly associated with both tumor staging and growth patterns; and 5) along with other ERV-env family members was regulated by methylation. We previously determined that Syncytin-1 could functionally mediate a steroid hormone proliferative response upon stimulation of epithelial EnCa cells with E2, tamoxifen or Progesterone [8, 9]. It is known that endometrial tumors produce approximately 58% higher E2, which could contribute to tumor proliferation through activation of Syncytin-1 [63]. In the present study, ERa transfected RL95-2 cells showed that luciferase was activated after E2 treatment via the ERV-W ERE, which was dependant on methylation. Interestingly, we found that tamoxifen activated the ERV-W LTR luciferase vector more than E2 in ERa transfected RL95-2- cells (E2: 1,567-fold; tamoxifen: 3,396-fold; unpublished data).

It is important to note that using IHC, 38% of premenopausal, lymph node negative women with breast cancer expressed Syncytin-1 protein and correlated with a positive prognostic indicator, probably due to cell-cell fusions between tumor and normal cells, leading to a loss of tumorigenic genes [35]. In contrast, Larsen et al. [36] showed that colorectal tumor epithelial cells were positive for Syncytin-1 expression, but the authors also found increased Syncytin-1 expression at the invasive growing tumor areas. In contrast to breast tumors, these findings associated significantly with a decreased overall survival of these colorectal carcinoma patients. Our previous findings showed multi-nucleated primary EnCa and cell-cell fusion of EnCa cell lines, which was regulated by cAMP signalling [8]. In this present investigation we demonstrated de-methylation and regulation of the CRE in primary EnCa tissues and luciferase assays in cell culture. Therefore, similar to the hypothesis of Lu and Kang [64] it is possible that cell-cell fusions between EnCa cells could result in selection of chromosomes with active oncogenes or deleted / mutated tumor suppressors leading to a more aggressive tumor.

## METHODS

### Patient clinical characteristics and pathological tissues

Handling of patients and tissues was approved by the Ethics Committee at the University of Erlangen-Nuremberg. All patients gave a written informed consent. Tissues were collected between 2002 and 2008, classified by the Institute of Pathology (University-Clinic Erlangen) and flash frozen in liquid nitrogen. Thirty-eight endometrioid EnCa tumors were grouped according to the Tumor – (Lymph) Nodes – Metastasis (TNM) classification stages T1a to T3b and differentiation growth patterns G1 (differentiated) to G3 (undifferentiated). T1a represents tumor localisation to the endometrium; T1b endometrial localisation with over 50% myometrial growth, T2 demonstrates tumor infiltration into the stroma of cervix uteri; and T3 and T4 with distant metastasis [65]. According to the above classifications our EnCa cohort represented T1a/G1 (n=8), T1a/G2 (n=4), T1a/G3 (n=3), T1b/G1 (n= 3), T1b/G2 (n=5), T1b/G3 (n= 2), T2/G1 (n=4), T2/G2 (n=5), T2/G3 (n=2), T3a/G3(n=1) and T3b/G3 (n=1) (mean age: 67.71 +/− 1.73 yr). Endometrial tissues free of carcinoma, polyp, hyperplasia and hyperproliferation from 29 postmenopausal patients were used as controls (7 patient-matched and 22 age-matched patients) (mean age: 67.62 +/−1.88 yr). Endometrial polyps were from 20 postmenopausal patients (mean age: 63.55 +/− 2.06 yr). Nine endometrial hyperplasias were classified as simple (n=6) and complex hyperplasia (n=3), with (n=2) and without atypia (n=7) (mean age: 56.1 +/− 4.14 yr).

### RNA isolation and absolute quantitative real time PCR (qPCR)

Total RNA was extracted from 30-50 mg of frozen tissues according to Strick et al. [8]. For expression analysis, RNA was pre-treated with DNase I (Sigma-Aldrich, Germany) and cDNA was generated with the High Capacity cDNA Kit (Applied Biosystems (ABI), Darmstadt, Germany) in a thermal cycler (ABI2720) for 2 hr at 37°C. Supplemental Table 1A shows specific env gene primers used for qPCR. Primers for Syncytin-2 and Syncytin-3, erv-3, envV1 and envV2 were according to Ruebner et al. [27]. Since envW2 is 93% similar to Syncytin-1 we designed new primers which distinguish between both env genes (suppl. Table 1A). The designed TF-primer for Syncytin-1 on 7q21.2 did not align to the env sequences of envW2 (Xq22.3) and other transcribed envW sequences on chromosomes 14q21.3, 15q21.3, 5q11.2, 6q21 and 17q21 [66], due to a 12 bp deletion of Syncytin-1 in this region. ERV env genes were amplified by qPCR from 40 ng of tissue cDNA with SYBR-green technology and analyzed with an ABI7300 (ABI, Darmstadt, Germany). cDNA was used to PCR amplify DNA fragments using primer sequences and then each env gene was cloned into TopoTA vectors (Invitrogen) (suppl. Table 1A). For qPCR analyses each cloned env gene with a known copy number was used as an external standard to generate a standard curve with a cycle threshold (C_T_) value against the log of amount of standard. Importantly, a similar PCR efficiency (over 97 %) between all env genes was needed for comparison. Similar standard curves of all env genes were obtained for the SYBR-green based qPCR with the following slopes and calculations (see suppl. Tab. 1B). Expression values were calculated as molecules (mol) per ng total RNA using a standard curve of each cloned env gene determined by real time PCR and calculated as mean +/− standard deviation of the mean (s.e.m.) according to Ruebner et al. [27]. All gene expressions were normalized to the co-amplified 18S-rRNA and one patient cDNA was used as an internal control.

### Immunolocalization of Syncytin-1, Syncytin-2 and erv-3 in EnCa, hyperplasia, polyps and normal endometrium using immunohistochemistry

Tissue samples were fixed in 10% formalin for 1 hr, washed several times with ethanol (70 -100%) for 5.5 hr and xylol (2.5 hr) and embedded into paraffin (2 hr). Hematoxylin/Eosin (HE) staining of 5μm tissue sections was performed by automation (Gemini, Shandon Varistain) following deparaffinization with xylol for 10 min, washed with ethanol and water and then stained with hematoxylin gill #3 (3 min) and eosin (20 sec). IHC stains were performed on tissue sections using the LSAB+HRP kit (Dako) according to the manufacturer's instructions. The following primary antibodies were used: Syncytin-1 (1:200, rabbit polyclonal from Imgenex, San Diego, CA, USA), Syncytin-2 (1:200, rabbit polyclonal from Abcam, Cambridge, UK) and erv-3 (1:1,000, goat polyclonal from Everest Biotech, Upper Heyford, UK).

### Analysis of the stop-codon of erv-3

In order to determine the incidence of the erv-3 stop codon [24] we designed a SNP-assay (ABI assay-by-design) with the following primer: 5' (VIC) CCGCTGACTCGTGCC (wild-type) and 5' (FAM) CGCTGACTCATGCC (mutant). Genomic DNA from EnCa and patient matched endometrium tissues were isolated according to Strissel et al. [67] and genotyped using both the wild-type, mutant primers and genotyping Master-mix (ABI) according to Binder et al [68].

### Bisulfite sequencing of ERV-W 5'LTR

Genomic DNA from control (n=10) and patient matched EnCa tissues (n=10) were isolated according to Strissel et al. [67], bisulfite treated with the EpiTect Bisulfite Kit (Qiagen) and the entire 5'LTR of ERV-W on chromosome 7q21.2 was amplified using specific primers 5'->3': Syn1UF (AGGATTAGTTGGATTTTTTAGGTTGA), SynMF (TAGGATTAGTTGGATTTTTTAGGTC), Syn1R (CCCAAATAACCTCACACCTA). Amplified fragments were cloned into the pSC-A vector (Stratagene) and for each tissue a minimum of 10 clones were sequenced using ABI3730 DNA Analyzer (ABI). For quality control of each bisulfite treated DNA all cloned fragments had to represent a minimum of <4% in cytosine content. The mean methylation of each of the 20 CpG's in the 5'LTR (Fig. 5) was determined for each patient pair in per cent (0% = no; 100% = full methylation). A mean methylation grade of all 10 controls and 10 EnCa was also calculated.

### Culturing of cell lines

We routinely verify our EnCa cell lines by a cell authentication service (LGC standards, Middlesex, UK), which matched our RL95-2 cell line for all analysed loci as RL95-2 from ATCC (CRL-1671). In addition, we verify our EnCa cell lines by immuno-blotting for the steroid hormone receptor ERα. However, over time we observed that one of our culture passages of RL95-2-ERα(+) cells, although verified by LGC standards as the RL95-2 cell line, had spontaneously lost the ERα expression, as confirmed by immunoblotting with an ERa antibody (1:1,000) (Cell Signaling, Danvers, USA) (Fig. 6C). For cell culturing, RL95-2-ERα(-) cells and the endometrial cell line HEC1A, which is also ERα(-), were maintained in RPMI, 10% FCS, non-essential amino acids and insulin, transferrin and selenium at 5% CO_2_ at 37^°^C. Treatment of cell lines with de-methylating agents 5-Aza-2'-deoxycytidine (AzadC) (1.5 μM) or RG108 (20 μM) was performed for 4 days.

### Cell Transfection and luciferase assays

Four fragments from the ERV-W 5'LTR promoter and upstream regulating regions (URE) (URE/U3/R/U5, 5'LTR (=U3/R/U5), U3 region containing the estrogen receptor response element (ERE) and the U5-region) were cloned into the luciferase plasmid pGL3basic (Promega) [30]. The 5'LTR and the U3 plasmids were methylated according to Gimenez et al. [40]. Luciferase assays (Roche) were performed with or without 17-b-estradiol (E2) or Forskolin (FK) in 6 independent transfections (3 μg vector and a transfection control with 2 μg of the β-Galactosidase expression-plasmid pSVβGal (Promega)) of the EnCa cell line RL95-2 using the JetPi transfection reagent (PeqLab, Erlangen) according to manufacturer's instructions. In all luciferase experiments involving E2 inductions, a CMV-ERa containing vector (HEGO-CMV-ERα, a kind gift from Dr. R.X. Song, University of Virginia, USA) was co-transfected along with the luciferase vector into RL95-2-ERα(-). Immunoblotting was performed to confirm transfection efficiency using an ERa antibody (1:1,000) (Cell Signaling, Danvers, USA). After 16 hr 5% CTS was added to the cells along with 50 nM E2. Statistics were performed with a minimum of 6 measurements per plasmid. Luciferase analysis was performed 48 hr post-transfection and all values for each construct were normalized to pGL3basic and ß-galactosidase.

### Statistical analyses

The non-parametric Mann-Whitney-U test for independent samples was performed using IBM SPSS Statistics 19 (IBM, Germany). For all tests a P value: <0.05 was considered as statistically significant. For each mean value, a standard error of the mean (s.e.m.) was calculated using IBM SPSS Statistics 19.

## Supplementary Tables



## References

[1] Jemal A, Bray F, Center MM, Ferlay J, Ward E, Forman D (2011). Global cancer statistics. CA Cancer J Clin.

[2] Sherman ME (2000). Theories of endometrial carcinogenesis: a multidisciplinary approach. Mod Pathol.

[3] Lacey JV, Chia VM (2009). Endometrial hyperplasia and the risk of progression to carcinoma. Maturitas.

[4] Horn LC, Schnurrbusch U, Bilek K, Einenkel J (2001). Endometrial hyperplasia: The risk of progression to carcinoma in a series of 538 cases. Geburtshilfe Und Frauenheilkunde.

[5] Ben-Arie A, Goldchmit C, Laviv Y, Levy R, Caspi B, Huszar M, Dgani R, Hagay Z (2004). The malignant potential of endometrial polyps. Eur J Obstet Gynecol Reprod Biol.

[6] Wethington SL, Herzog TJ, Burke WM, Sun X, Lerner JP, Lewin SN, Wright JD (2011). Risk and predictors of malignancy in women with endometrial polyps. Ann Surg Oncol.

[7] Okuda T, Sekizawa A, Purwosunu Y, Nagatsuka M, Morioka M, Hayashi M, Okai T (2010). Genetics of endometrial cancers. Obstet Gynecol Int.

[8] Strick R, Ackermann S, Langbein M, Swiatek J, Schubert SW, Hashemolhosseini S, Koscheck T, Fasching PA, Schild RL, Beckmann MW, Strissel PL (2007). Proliferation and cell-cell fusion of endometrial carcinoma are induced by the human endogenous retroviral Syncytin-1 and regulated by TGF-beta. J Mol Med (Berl).

[9] Strissel PL, Ellmann S, Loprich E, Thiel F, Fasching PA, Stiegler E, Hartmann A, Beckmann MW, Strick R (2008). Early aberrant insulin-like growth factor signaling in the progression to endometrial carcinoma is augmented by tamoxifen. Int J Cancer.

[10] Maxwell GL, Chandramouli GV, Dainty L, Litzi TJ, Berchuck A, Barrett JC, Risinger JI (2005). Microarray analysis of endometrial carcinomas and mixed mullerian tumors reveals distinct gene expression profiles associated with different histologic types of uterine cancer. Clin Cancer Res.

[11] Risinger JI, Maxwell GL, Chandramouli GV, Jazaeri A, Aprelikova O, Patterson T, Berchuck A, Barrett JC (2003). Microarray analysis reveals distinct gene expression profiles among different histologic types of endometrial cancer. Cancer Res.

[12] Bannert N, Kurth R (2004). Retroelements and the human genome: new perspectives on an old relation. Proc Natl Acad Sci U S A.

[13] Stoye JP (2012). Studies of endogenous retroviruses reveal a continuing evolutionary saga. Nat Rev Microbiol.

[14] Tristem M (2000). Identification and characterization of novel human endogenous retrovirus families by phylogenetic screening of the human genome mapping project database. J Virol.

[15] Benit L, Calteau A, Heidmann T (2003). Characterization of the low-copy HERV-Fc family: evidence for recent integrations in primates of elements with coding envelope genes. Virology.

[16] Taruscio D, Floridia G, Zoraqi GK, Mantovani A, Falbo V (2002). Organization and integration sites in the human genome of endogenous retroviral sequences belonging to HERV-E family. Mamm Genome.

[17] Costas J (2002). Characterization of the intragenomic spread of the human endogenous retrovirus family HERV-W. Mol Biol Evol.

[18] Hughes JF, Coffin JM (2001). Evidence for genomic rearrangements mediated by human endogenous retroviruses during primate evolution. Nat Genet.

[19] Whitelaw E, Martin DI (2001). Retrotransposons as epigenetic mediators of phenotypic variation in mammals. Nat Genet.

[20] de Parseval N, Lazar V, Casella JF, Benit L, Heidmann T (2003). Survey of human genes of retroviral origin: identification and transcriptome of the genes with coding capacity for complete envelope proteins. J Virol.

[21] Repaske R, Steele PE, O'Neill RR, Rabson AB, Martin MA (1985). Nucleotide sequence of a full-length human endogenous retroviral segment. J Virol.

[22] Turbeville MA, Rhodes JC, Hyams DM, Distler CM, Steele PE (1997). Characterization of a putative retroviral env-related human protein. Pathobiology.

[23] Roebke C, Wahl S, Laufer G, Stadelmann C, Sauter M, Mueller-Lantzsch N, Mayer J, Ruprecht K (2010). An N-terminally truncated envelope protein encoded by a human endogenous retrovirus W locus on chromosome Xq22.3. Retrovirology.

[24] de Parseval N, Heidmann T (1998). Physiological knockout of the envelope gene of the single-copy ERV-3 human endogenous retrovirus in a fraction of the Caucasian population. J Virol.

[25] Blond JL, Lavillette D, Cheynet V, Bouton O, Oriol G, Chapel-Fernandes S, Mandrand B, Mallet F, Cosset FL (2000). An envelope glycoprotein of the human endogenous retrovirus HERV-W is expressed in the human placenta and fuses cells expressing the type D mammalian retrovirus receptor. J Virol.

[26] Mi S, Lee X, Li X, Veldman GM, Finnerty H, Racie L, LaVallie E, Tang XY, Edouard P, Howes S, Keith JC, McCoy JM (2000). Syncytin is a captive retroviral envelope protein involved in human placental morphogenesis. Nature.

[27] Ruebner M, Strissel PL, Langbein M, Fahlbusch F, Wachter DL, Faschingbauer F, Beckmann MW, Strick R (2010). Impaired cell fusion and differentiation in placentae from patients with intrauterine growth restriction correlate with reduced levels of HERV envelope genes. J Mol Med (Berl).

[28] Blaise S, de Parseval N, Heidmann T (2005). Functional characterization of two newly identified Human Endogenous Retrovirus coding envelope genes. Retrovirology.

[29] Langbein M, Strick R, Strissel PL, Vogt N, Parsch H, Beckmann MW, Schild RL (2008). Impaired cytotrophoblast cell-cell fusion is associated with reduced Syncytin and increased apoptosis in patients with placental dysfunction. Mol Reprod Dev.

[30] Ruebner M, Langbein M, Strissel PL, Henke C, Schmidt D, Goecke TW, Faschingbauer F, Schild RL, Beckmann MW, Strick R (2012). Regulation of the human endogenous retroviral Syncytin-1 and cell-cell fusion by the nuclear hormone receptors PPARgamma/RXRalpha in placentogenesis. J Cell Biochem.

[31] Herbst H, Kuhler-Obbarius C, Lauke H, Sauter M, Mueller-Lantzsch N, Harms D, Loning T (1999). Human endogenous retrovirus (HERV)-K transcripts in gonadoblastomas and gonadoblastoma-derived germ cell tumours. Virchows Arch.

[32] Buscher K, Trefzer U, Hofmann M, Sterry W, Kurth R, Denner J (2005). Expression of human endogenous retrovirus K in melanomas and melanoma cell lines. Cancer Res.

[33] Wang-Johanning F, Frost AR, Johanning GL, Khazaeli MB, LoBuglio AF, Shaw DR, Strong TV (2001). Expression of human endogenous retrovirus k envelope transcripts in human breast cancer. Clin Cancer Res.

[34] Wang-Johanning F, Liu J, Rycaj K, Huang M, Tsai K, Rosen DG, Chen DT, Lu DW, Barnhart KF, Johanning GL (2007). Expression of multiple human endogenous retrovirus surface envelope proteins in ovarian cancer. Int J Cancer.

[35] Bjerregaard B, Holck S, Christensen IJ, Larsson LI (2006). Syncytin is involved in breast cancer-endothelial cell fusions. Cell Mol Life Sci.

[36] Larsen JM, Christensen IJ, Nielsen HJ, Hansen U, Bjerregaard B, Talts JF, Larsson LI (2009). Syncytin immunoreactivity in colorectal cancer: potential prognostic impact. Cancer Lett.

[37] Sun Y, Ouyang DY, Pang W, Tu YQ, Li YY, Shen XM, Tam SC, Yang HY, Zheng YT (2010). Expression of syncytin in leukemia and lymphoma cells. Leuk Res.

[38] Lavie L, Kitova M, Maldener E, Meese E, Mayer J (2005). CpG methylation directly regulates transcriptional activity of the human endogenous retrovirus family HERV-K(HML-2). J Virol.

[39] Reiss D, Zhang Y, Mager DL (2007). Widely variable endogenous retroviral methylation levels in human placenta. Nucleic Acids Res.

[40] Gimenez J, Montgiraud C, Oriol G, Pichon JP, Ruel K, Tsatsaris V, Gerbaud P, Frendo JL, Evain-Brion D, Mallet F (2009). Comparative methylation of ERVWE1/syncytin-1 and other human endogenous retrovirus LTRs in placenta tissues. DNA Res.

[41] Matouskova M, Blazkova J, Pajer P, Pavlicek A, Hejnar J (2006). CpG methylation suppresses transcriptional activity of human syncytin-1 in non-placental tissues. Exp Cell Res.

[42] de Parseval N, Casella J, Gressin L, Heidmann T (2001). Characterization of the three HERV-H proviruses with an open envelope reading frame encompassing the immunosuppressive domain and evolutionary history in primates. Virology.

[43] Hirose Y, Takamatsu M, Harada F (1993). Presence of env genes in members of the RTVL-H family of human endogenous retrovirus-like elements. Virology.

[44] Lindeskog M, Mager DL, Blomberg J (1999). Isolation of a human endogenous retroviral HERV-H element with an open env reading frame. Virology.

[45] Mager DL, Henthorn PS (1984). Identification of a retrovirus-like repetitive element in human DNA. Proc Natl Acad Sci U S A.

[46] Wilkinson DA, Freeman JD, Goodchild NL, Kelleher CA, Mager DL (1990). Autonomous expression of RTVL-H endogenous retroviruslike elements in human cells. J Virol.

[47] Mangeney M, de Parseval N, Thomas G, Heidmann T (2001). The full-length envelope of an HERV-H human endogenous retrovirus has immunosuppressive properties. J Gen Virol.

[48] Cohen M, Kato N, Larsson E (1988). ERV3 human endogenous provirus mRNAs are expressed in normal and malignant tissues and cells, but not in choriocarcinoma tumor cells. J Cell Biochem.

[49] Rote NS, Chakrabarti S, Stetzer BP (2004). The role of human endogenous retroviruses in trophoblast differentiation and placental development. Placenta.

[50] Lin L, Xu B, Rote NS (2000). The cellular mechanism by which the human endogenous retrovirus ERV-3 env gene affects proliferation and differentiation in a human placental trophoblast model, BeWo. Placenta.

[51] Gimenez J, Montgiraud C, Pichon JP, Bonnaud B, Arsac M, Ruel K, Bouton O, Mallet F (2010). Custom human endogenous retroviruses dedicated microarray identifies self-induced HERV-W family elements reactivated in testicular cancer upon methylation control. Nucleic Acids Res.

[52] Wang-Johanning F, Frost AR, Jian B, Azerou R, Lu DW, Chen DT, Johanning GL (2003). Detecting the expression of human endogenous retrovirus E envelope transcripts in human prostate adenocarcinoma. Cancer.

[53] Wang-Johanning F, Frost AR, Jian B, Epp L, Lu DW, Johanning GL (2003). Quantitation of HERV-K env gene expression and splicing in human breast cancer. Oncogene.

[54] Wang-Johanning F, Radvanyi L, Rycaj K, Plummer JB, Yan P, Sastry KJ, Piyathilake CJ, Hunt KK, Johanning GL (2008). Human endogenous retrovirus K triggers an antigen-specific immune response in breast cancer patients. Cancer Res.

[55] Yi JM, Kim HS (2007). Molecular phylogenetic analysis of the human endogenous retrovirus E (HERV-E) family in human tissues and human cancers. Genes Genet Syst.

[56] Wang-Johanning F, Rycaj K, Plummer JB, Li M, Yin B, Frerich K, Garza JG, Shen J, Lin K, Yan P, Glynn SA, Dorsey TH, Hunt KK, Ambs S, Johanning GL (2012). Immunotherapeutic potential of anti-human endogenous retrovirus-K envelope protein antibodies in targeting breast tumors. J Natl Cancer Inst.

[57] Blaise S, de Parseval N, Benit L, Heidmann T (2003). Genomewide screening for fusogenic human endogenous retrovirus envelopes identifies syncytin 2, a gene conserved on primate evolution. Proc Natl Acad Sci U S A.

[58] Mangeney M, Renard M, Schlecht-Louf G, Bouallaga I, Heidmann O, Letzelter C, Richaud A, Ducos B, Heidmann T (2007). Placental syncytins: Genetic disjunction between the fusogenic and immunosuppressive activity of retroviral envelope proteins. Proc Natl Acad Sci U S A.

[59] Iguchi-Ariga SM, Schaffner W (1989). CpG methylation of the cAMP-responsive enhancer/promoter sequence TGACGTCA abolishes specific factor binding as well as transcriptional activation. Genes Dev.

[60] Salvesen HB, Stefansson I, Kretzschmar EI, Gruber P, MacDonald ND, Ryan A, Jacobs IJ, Akslen LA, Das S (2004). Significance of PTEN alterations in endometrial carcinoma: a population-based study of mutations, promoter methylation and PTEN protein expression. Int J Oncol.

[61] Banno K, Yanokura M, Susumu N, Kawaguchi M, Hirao N, Hirasawa A, Tsukazaki K, Aoki D (2006). Relationship of the aberrant DNA hypermethylation of cancer-related genes with carcinogenesis of endometrial cancer. Oncol Rep.

[62] Stengel S, Fiebig U, Kurth R, Denner J (2010). Regulation of human endogenous retrovirus-K expression in melanomas by CpG methylation. Genes Chromosomes Cancer.

[63] Berstein LM, Tchernobrovkina AE, Gamajunova VB, Kovalevskij AJ, Vasilyev DA, Chepik OF, Turkevitch EA, Tsyrlina EV, Maximov SJ, Ashrafian LA, Thijssen JH (2003). Tumor estrogen content and clinico-morphological and endocrine features of endometrial cancer. J Cancer Res Clin Oncol.

[64] Lu X, Kang Y (2009). Cell fusion as a hidden force in tumor progression. Cancer Res.

[65] Horn LC, Beckmann MW, Beller A, Schmidt D, Ulrich U, Hantschmann P, Wittekind C (2010). [Changes in the TNM classification of gynecological tumors]. Pathologe.

[66] Laufer G, Mayer J, Mueller BF, Mueller-Lantzsch N, Ruprecht K (2009). Analysis of transcribed human endogenous retrovirus W env loci clarifies the origin of multiple sclerosis-associated retrovirus env sequences. Retrovirology.

[67] Strissel PL, Swiatek J, Oppelt P, Renner SP, Beckmann MW, Strick R (2007). Transcriptional analysis of steroid hormone receptors in smooth muscle uterine leiomyoma tumors of postmenopausal patients. J Steroid Biochem Mol Biol.

[68] Binder H, Dittrich R, Hager I, Muller A, Oeser S, Beckmann MW, Hamori M, Fasching PA, Strick R (2008). Association of FSH receptor and CYP19A1 gene variations with sterility and ovarian hyperstimulation syndrome. Reproduction.

[69] Mayer J, Blomberg J, Seal RL (2011). A revised nomenclature for transcribed human endogenous retroviral loci. Mobile DNA.

