# Supplementary material for: Reactivation of codogenic endogenous retroviral (ERV) envelope genes in human endometrial carcinoma and prestages: Emergence of new molecular targets
Source: Oncotarget. 2012 Oct 13;3(10):1204–19. doi: 10.18632/oncotarget.679 (PMC3717959; doi:10.18632/oncotarget.679)
Supplement: Supplementary file 1 [file oncotarget-03-1204-s001.pdf]

Reactivation of codogenic endogenous retroviral (ERV) envelope genes in human endometrial carcinoma and prestages: Emergence of new molecular targets – Strissel et al

Supplemental Table 1a: Primer for cloning and qPCR of env genes.

genes	TF and BR cloning primers : 5´→ 3´	TF and BR qPCR primers : 5´→ 3´
Syncytin-1	[29]	ATGGAGCCCAAGATGCAG<br>AGATCGTGGGCTAGCAG
Syncytin-2	[29]	TTGGGTAAATCAATCAGGAAAAGT<br>GTAAACTGGAGGCTTGATTTAGGA
Syncytin-3	[29]	TTAGCCACAAATTCTGGGATAACT<br>AGAGGTAACAATAGAGGCCATGAG
erv-3	[29]	AACTAATGCCCCAAGATAATTTCA<br>TTAAGAACCAGATGCTCTGACTTG
envV1	[29]	GTGGCTCCATAACTTTGGAAAA<br>TAAGTGCAGCTGGTCCCAGTA
envV2	[29]	TGTGTCTCTTCTAGGATAAAGCAATTT<br>AGGGGGAGATGTGCTTATAGGT
envT	GACGCTAGCTGTAGAGATACTTACCAGT<br>TTAAGTGTGGCTTCTACTCCTAATCCTA	AGGATTTGATGTTGGGACTATGTT<br>GGTGTTTCTGGAATATAGGGTCAC
envRb	GTCCAGACTTCCAGACCTCAGAACACTCA<br>GTGCATATAATACACTTCAGCATTCAT	CACTAAGGGACACTTAAGCCAGAT<br>AGTATAGTATGTCGGCACTGTCCA
envK1-6	GACCTGAATTCCAGTCTAACAGTTCCT<br>CGATGTTCTAAGCTCATGAGTCTGTCT	TTTGACTGAAGTATTAAAAGGTGT<br>GTGACTGCAATTAATCCCATAATC
envH1-3	TATCTCCACCACACTATCAACCTTACCCATTC<br>AAGTGTCTTCCTAAGCAATAATTACTGCT	TCCCCAGACATTTCACATCAG<br>GAGCGGCCACAGGAATACTA
envE	CAGTGTATTCATCAGGTCACCGAGGTAGGACAG<br>AGTTCTAGTGGGTTACAGTGACCAGCAGCAC	ACTGGCCTTTTCCTAGGTGATAC<br>TACTATTAATGGCTGCACAAGCA
envFc1	CTGACATGGTCACCTATGATGACAAGGCTG<br>TGTAGAGAGGCTAAGGATTCGGCTGAGGCT	GCTACACCACTCCTAACTCATCCT<br>TTGTAAGGGTGAAGTTACACCAGA
envFc2	TGTTCTATGTCAATACCTCCACTGGAGGAC<br>TAGCGTGGAGTTACACCAGAAGTAGCCTCT	CTCCATTAGTAGCAGTTCCTCTCC<br>GAGAATAGTGGGACCTGTCCTTT
envW2	[24]	GCTACAAATGGTTCTTCAAATGGAG<br>ACAAGGGCTAGCAGGCT

Supplemental Table 1B: Slope (γ), PCR amplification efficiency (correlation coefficient) (R^2) and intercept (t) of standard curves of cloned env genes in qPCR.

**HERV**	**gene**	**γ:**	**R^2:**	**t:**
-W	Syncytin-1	-3.1721	0.9919	31.269
-FRD	Syncytin-2	-3.0772	0.9953	31.7325
-P(b)	Syncytin-3	-3.4553	0.9776	36.012
-R	erv-3	-3.2032	0.9933	35.328
-K1-6	envK1-6	-3.1379	0.9987	34.887
-V1	envV1	-3.2252	0.9890	33.247
-V2	envV2	-3.1490	0.9978	32.721
-E4-1	envE	-3.1361	0.9865	26.9127
-H1-3	envH1-3 (p59,60,62)	-3.2946	0.9905	27.6260
-T	envT	-3.4772	0.9931	34.7865
-R(b)	envRb	-3.3099	0.9977	34.198
-Fc1	envFc1	-3.289	0.9988	33.742
-Fc2	envFc2	-3.4686	0.9987	39.6515
-W2	envW2	-3.5424	0.9886	33.62

Supplemental Table 2: Gene expression by qPCR and P-values.

	control (n= 31)	polyp (n=20)	hyperplasia (n=9)	EnCa (n=38)	hierachical expression
genes	mol/ng ± sem	mol/ng ± sem	mol/ng ± sem	mol/ng ± sem	low →high
Syncytin -1	18.52 ± 6.67	**88.54 ± 34.59 P: 0.0025**	**77.7 ± 21.8 P: 1.10 E-4**	**236.76 ± 77.55 P: 6.72 E-8**	control > hyper > polyp > EnCa
Syncytin -2	1.44 ± 0.30	**2.82 ± 0.73 P: 0.049**	**8.95 ± 4.94 P: 0.0107**	**9.90 ± 2.80 P: 5.71 E-4**	control > polyp > hyper > EnCa
Syncytin -3	2.04 ± 0.53	**5.43 ± 1.63 P: 0.0044**	**13.14 ± 7.54 P: 0.0016**	**15.04 ± 3.38 P: 3.59 E-5**	control > polyp > hyper > EnCa
erv-3	30.47 ± 7.25	**134.54 ± 38.45 P: 5.15 E-4**	**246.68 ± 97.39 P: 0.0021**	**82.95 ± 19.48 P: 4.10 E-5**	control > EnCa > polyp > hyper
envK1-6	3.32 ± 0.92	**11.82 ± 3.74 P: 0.017**	15.41 ± 10.88 P: 0.276	**41.61 ± 17.27 P: 6.06 E-6**	control > polyp > hyper > EnCa
envT	28.27 ± 10.06	108.66 ± 51.40 P: 0.079	**72.09 ± 22.74 P: 0.0052**	**195.14 ± 68.85 P: 1.34 E-4**	control > hyper > polyp > EnCa
envRb	0.74 ± 0.31	**18.42 ± 11.42 P: 0.0372**	**6.35 ± 3.87 P: 0.033**	**6.03 ± 2.00 P: 0.0062**	control > EnCa > hyper > polyp
envFc1	1.03 ± 0.40	**8.43 ± 5.03 P: 0.048**	4.06 ± 1.93 P: 0.052	**12.13 ± 4.40 P: 4.02 E-4**	control > hyper > polyp > EnCa
envFc2	8.31 ± 2.13	45.52 ± 23.59 P: 0.140	**26.03 ± 8.3 P: 0.00131**	**67.80 ± 17.27 P: 8.06 E-6**	control > hyper > polyp > EnCa

envH1-3	126.01 ± 43.13	711.17 ± 400.09<br>P: 0.276	**479.25 ± 192.61<br>P: 0.0015**	**1,378.41 ± 596.86<br>P: 3.99 E-5**	control > hyper > polyp > EnCa
envV1	1.15 ± 0.59	**10.64 ± 6.42<br>P: 0.0094**	**3.73 ± 1.65<br>P: 0.0067**	**10.64 ± 4.15<br>P: 0.0019**	control > hyper > polyp = EnCa
envV2	0.44 ± 0.27	**7.80 ± 4.90<br>P: 0.042**	1.40 ± 0.50<br>P: 0.056	**7.04 ± 2.80<br>P: 5.45 E-4**	control > hyper > EnCa > polyp
envE	18.51 ± 6.59	16.86 ± 6.22<br>P: 0.396	**239.61 ± 139.65<br>P: 0.0134**	**212.95 ± 122.15<br>P: 1.64 E-7**	polyp > control > EnCa > hyper
envW2	23.05 ± 6.91	**123.26 ± 48.85<br>P: 0.002**	**108.61 ± 37.79<br>P: 5.43 E-4**	**234.8 ± 72.19<br>P: 8.03 E-7**	control > hyper > polyp > EnCa

		pT1a<br>n= 15	pT1b<br>n= 10	pT2<br>n= 11	G1<br>n= 15	G2<br>n= 14	G3<br>n= 9
Syncytin-1	mol/ng	357.92	107.84	453.03	220.63	228.95	587.39
	+/- SEM	108.46	60.22	227.96	69.38	114.56	261.17
	P:		0.035			0.027	
Syncytin-2	mol/ng	8.69	4.65	17.52	12.50	5.59	12.31
	+/- SEM	3.51	2.44	7.96	5.63	2.06	6.72
	P:		0.019				
Syncytin-3	mol/ng	15.31	12.09	18.74	17.07	7.71	23.07
	+/- SEM	4.70	5.69	8.57	4.90	4.03	9.63
erv-3	mol/ng	101.81	67.75	73.61	36.56	123.94	96.52
	+/- SEM	47.18	20.04	11.95	5.02	49.38	19.49

Supplemental Table 3: Gene expression of env genes by qPCR according to clinical categories.

	P:				0.0178 (G1:G2)	0.025 (G1:G3)	
envK1-6	mol/ng	25.44	16.71	91.56	30.30	16.99	98.77
	+/- SEM	8.04	9.68	57.08	9.92	7.86	69.52
envT	mol/ng	176.45	125.74	302.40	175.88	58.86	439.25
	+/- SEM	47.64	61.46	226.42	57.80	18.62	266.13
	P:					0.023	
envRb	mol/ng	10.23	0.83	5.39	9.33	2.10	6.68
	+/- SEM	4.18	0.69	3.54	4.28	1.33	3.96
	P:	0.0096					
envFc1	mol/ng	9.09	4.27	24.42	10.66	10.82	16.63
	+/- SEM	3.56	3.35	13.84	4.06	8.80	11.48
envFc2	mol/ng	65.79	54.34	83.75	79.38	31.22	105.42
	+/- SEM	17.46	35.44	46.23	27.40	10.86	53.60
	P:					0.027	
envH1-3	mol/ng	1,143.73	619.22	2,471.02	1,334.40	244.43	3,215.73
	+/- SEM	443.92	481.43	1,947.69	519.76	85.06	2,340.54
	P:					0.032	
envV1	mol/ng	10.23	6.68	15.79	13.12	1.84	20.20
	+/- SEM	3.97	6.06	12.48	5.26	1.07	15.04
	P:				0.049		
envV2	mol/ng	5.83	3.57	10.28	7.29	1.11	15.88
	+/- SEM	2.67	3.10	8.44	3.18	0.64	10.35
envE	mol/ng	118.32	53.33	504.11	127.31	50.42	608.50
	+/- SEM	26.62	19.36	420.56	31.95	12.49	511.64
	P:				0.038		
envW2	mol/ng	250.79	160.69	299.58	276.13	82.13	403.42
	+/- SEM	74.87	113.25	210.27	96.81	26.45	253.44

Only statistical significances (P<0.05) are shown.
